# Transcriptomic dataset of RAW264.7 murine macrophages pretreated with 9-methoxycanthin-6-one under poly(I:C)-TLR3 stimulation

**DOI:** 10.1016/j.dib.2025.112445

**Published:** 2026-01-07

**Authors:** Trang Thu Tran, Huyen Minh Thi Ta, Duc Hoang Le, Duong Huy Nguyen, Nam Trung Nguyen

**Affiliations:** a Institute of Biology (IB), Vietnam Academy of Science and Technology (VAST), Hanoi, 100000, Vietnam; b Graduate University of Science and Technology (GUST), Vietnam Academy of Science and Technology (VAST), Hanoi, 100000, Vietnam

**Keywords:** RNA-seq, Alkaloid compounds, Antiviral activity, Immune regulation, Illumina sequencing

## Abstract

This dataset presents RNA sequencing (RNA-seq) data from RAW264.7 murine macrophages pretreated with 9-methoxycanthin-6-one, a canthin-6-one–type alkaloid isolated from *Eurycoma longifolia* Jack, and subsequently stimulated with polyinosinic:polycytidylic acid [poly(I:C)], a synthetic double-stranded RNA analog that activates TLR3-mediated antiviral signaling. RAW264.7 cells were pretreated with 9-methoxycanthin-6-one (30 µM) for 30 min and then exposed to poly(I:C) (20 µg/mL) for 6 h. Total RNA was extracted, quality-checked, and sequenced on the Illumina platform to generate paired-end reads. Differential expression analysis and functional annotation were performed to profile genes responsive to 9-methoxycanthin-6-one treatment under poly(I:C) stimulation. The dataset includes normalized expression matrices, lists of upregulated and downregulated genes, and pathway enrichment outputs in standard formats. These data provide a reference resource for understanding the transcriptomic responses of macrophages to natural alkaloid treatment during viral-mimetic immune activation. The dataset can be reused to compare host antiviral transcriptional responses across TLR3-related pathways, evaluate macrophage activation markers, or integrate with other *E. longifolia* bioactive compounds.

Specifications Table

**Table:** 

Subject	Biology
Specific subject area	Natural product transcriptomics; macrophage immune response; polyinosinic:polycytidylic acid (poly(I:C))- Toll-like receptor 3 (TLR3) pathway; RNA sequencing (RNA-Seq) dataset
Type of data	Table, Graph, Raw, Analysed, Processed.
Data collection	RAW264.7 macrophages were treated with poly(I:C) (20 µg/mL) alone or combined with 9-methoxycanthin-6-one (30 µM) for 6 h. Total RNA was extracted, and libraries were prepared using the Illumina TruSeq Stranded mRNA protocol. Sequencing was performed on the Illumina NovaSeq 6000 platform (Illumina, USA), yielding approximately 650 million paired-end reads per sample
Data source location	Institute of Biology, Vietnam Academy of Science and Technology, Hanoi, Vietnam
Data accessibility	Repository name: NCBI’s Gene Expression Omnibus (GEO) Data identification number: BioProject PRJNA1348298, with BioSamples including SAMN52895578, SAMN52895940, and SAMN52941918. Direct URL to data: https://www.ncbi.nlm.nih.gov/bioproject/?term=PRJNA1348298
Related research article	None

## 1 Value of the Data

• This dataset is the first to provide high-quality RNA-Seq profiles of RAW264.7 murine macrophages treated with the natural alkaloid 9-methoxycanthin-6-one under poly(I:C)-TLR3 stimulation.
• Researchers can reuse these RNA-Seq data to perform comparative analyses of macrophage activation pathways, validate TLR3-related gene expression signatures, or integrate them into meta-analyses of host immune responses to 9-methoxycanthin-6-one.
• The dataset supports research in pharmacology and immunology. It can be combined with public transcriptomic datasets to explore structure–activity relationships of *E. longifolia*-derived canthin-6-one alkaloids or to identify shared transcriptional regulators between TLR3 and other innate immune pathways.

## 2 Background

The dataset was generated to explore the transcriptional effects of 9-methoxycanthin-6-one, a canthin-6-one–type alkaloid isolated from *Eurycoma longifolia* Jack, on antiviral immune signaling in macrophages. Previous research demonstrated that this compound suppresses inflammatory cytokines in bacterial lipopolysaccharide (LPS)-stimulated macrophages [1]. However, its influence on viral-mimetic immune activation through the polyinosinic:polycytidylic acid [poly(I:C)]–TLR3 pathway has not been characterized. To address this gap, we designed an RNA sequencing (RNA-Seq) experiment to capture the transcriptional response of RAW264.7 murine macrophages pretreated with 9-methoxycanthin-6-one and stimulated with poly(I:C). The dataset was compiled to provide a reference resource for identifying gene expression patterns associated with 9-methoxycanthin-6-one–mediated modulation of innate immune pathways. These data article complements ongoing research on the immunomodulatory actions of *E. longifolia* alkaloids by providing openly accessible transcriptomic data suitable for reuse, validation, and comparative pathway analysis in immune and pharmacological studies.

## 3 Data Description

The transcriptome dataset of RAW264.7 macrophages was generated from RNA sequencing data from three experimental conditions: an untreated control, cells stimulated with 20 µg/mL poly(I:C) for 6 hours, and cells pretreated with 30 µM 9-methoxycanthin-6-one (9MC6) followed by poly(I:C) stimulation. Sequencing on Illumina NovaSeq 6000 platform generated approximately 650 million paired-end reads (2 × 150 bp) per sample. The raw FASTQ files have been deposited in NCBI under accession numbers SAMN52895578, SAMN52895940, and SAMN52941918.

**Fig. 1:**
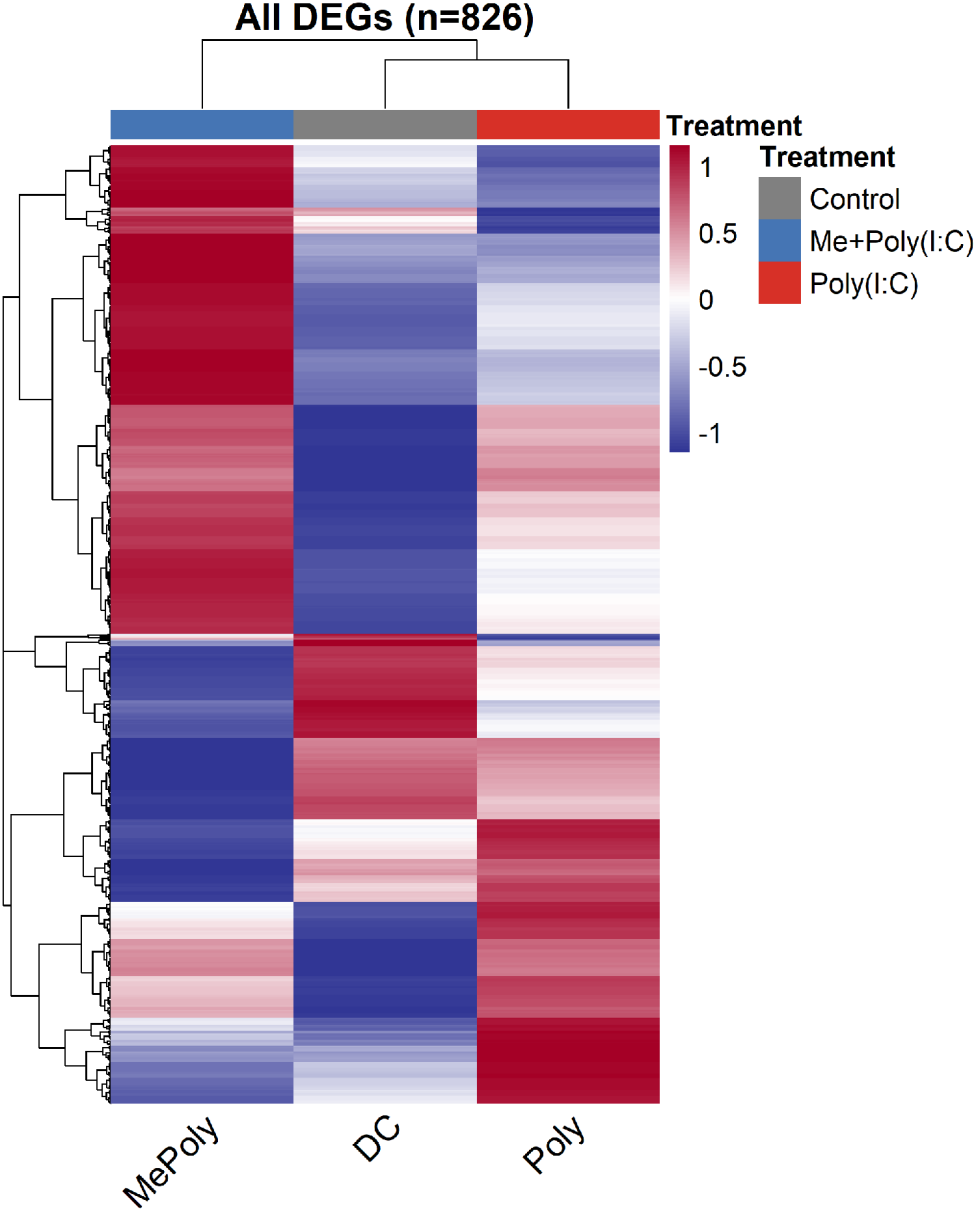
Hierarchical clustering heatmap reveals distinct expression signatures across treatment conditions. The heatmap displays normalized expression values (z-scores) for each gene across samples, with genes and samples clustered by Euclidean distance using complete linkage.

**Fig. 2:**
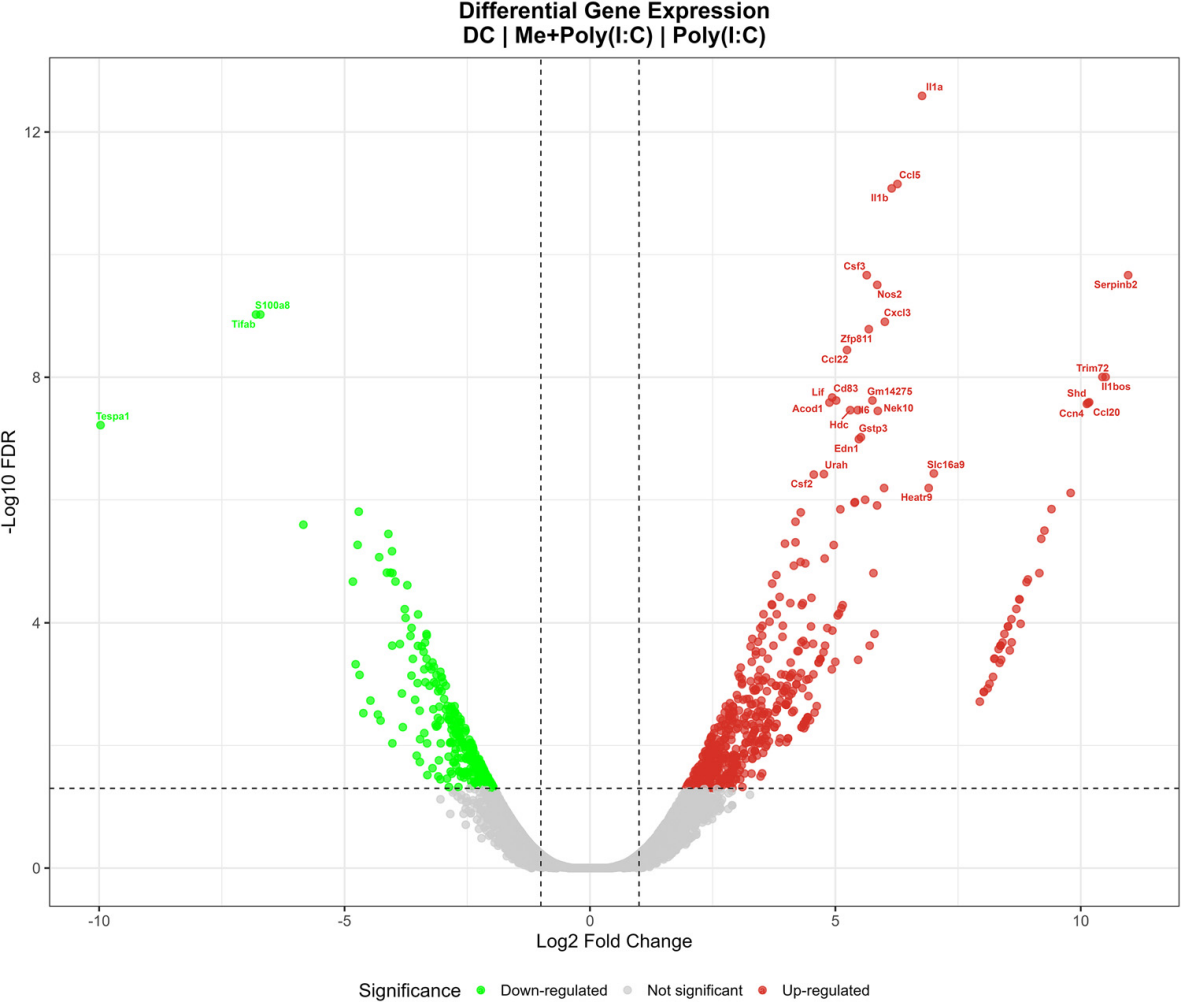
Volcano plot illustrating the magnitude and statistical significance of gene expression changes. Volcano plot displaying log2 fold changes (x-axis) and -log10(FDR) values (y-axis) for all detected genes across three pairwise comparisons: Poly vs Control, 9MC6+Poly vs Control, and 9MC6+Poly vs Poly. Red points indicate significantly upregulated genes (FDR < 0.05, log2FC > 1), green points represent downregulated genes (FDR < 0.05, log2FC < -1), and gray points denote non-significant genes. The vertical dashed lines mark the |log2FC| = 1 threshold, whereas the horizontal dashed line indicates the FDR = 0.05 significance cutoff.

**Fig. 3:**
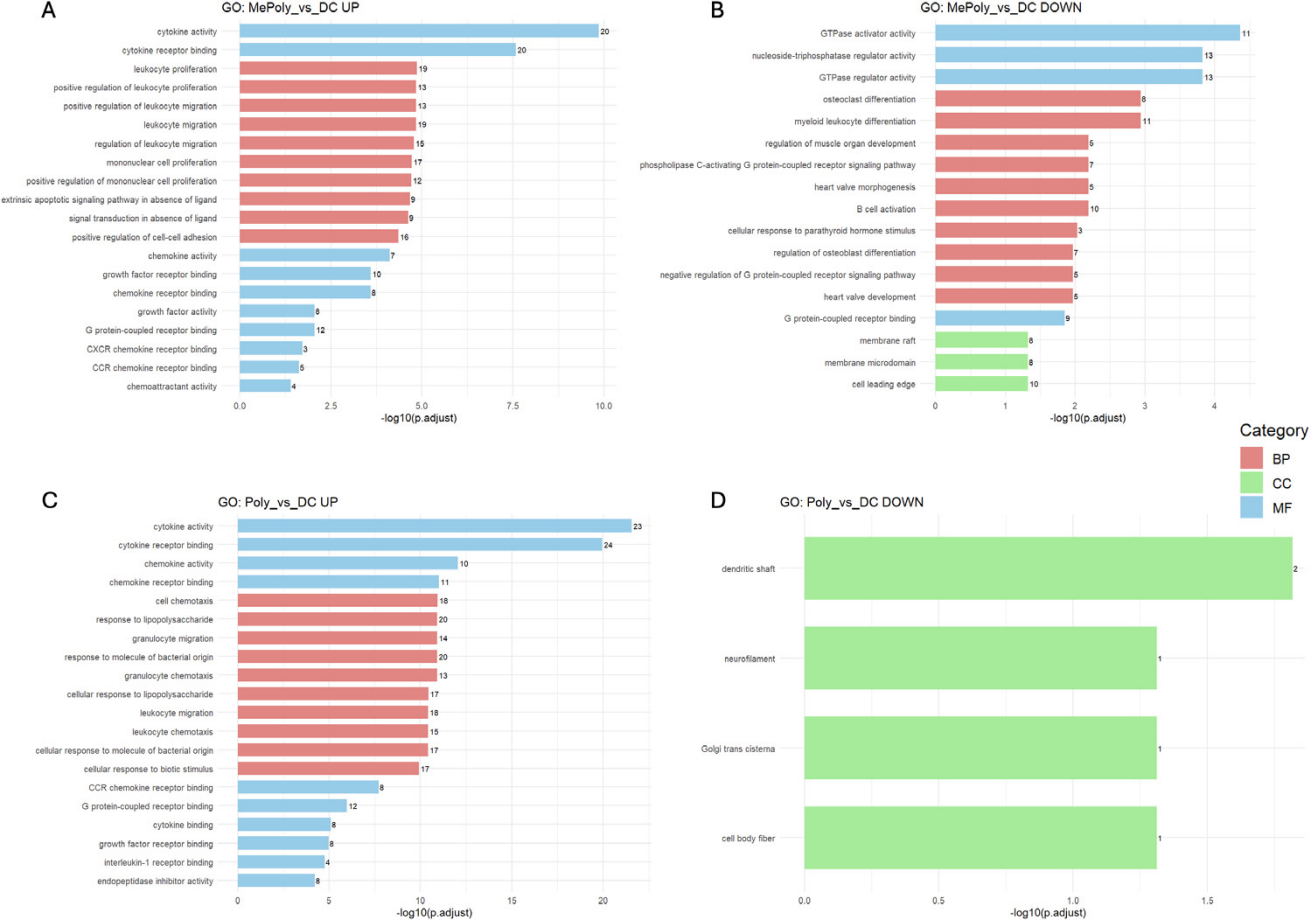
Gene Ontology enrichment analysis identifies overrepresented biological processes. Horizontal bar plots showing the top 10 enriched GO terms for upregulated (A, C) and downregulated (B, D) genes in 9MC6+Poly vs Control (A, B) and Poly vs Control (C, D) comparisons. The x-axis represents -log10 (p.adjust) values indicating statistical significance of enrichment. The mumbers at the ends of the bars indicate the number of genes mapped to each GO term. The colors distinguish the following GO categories: red (Biological Process), blue (Molecular Function), and green (Cellular Component).

**Fig. 4:**
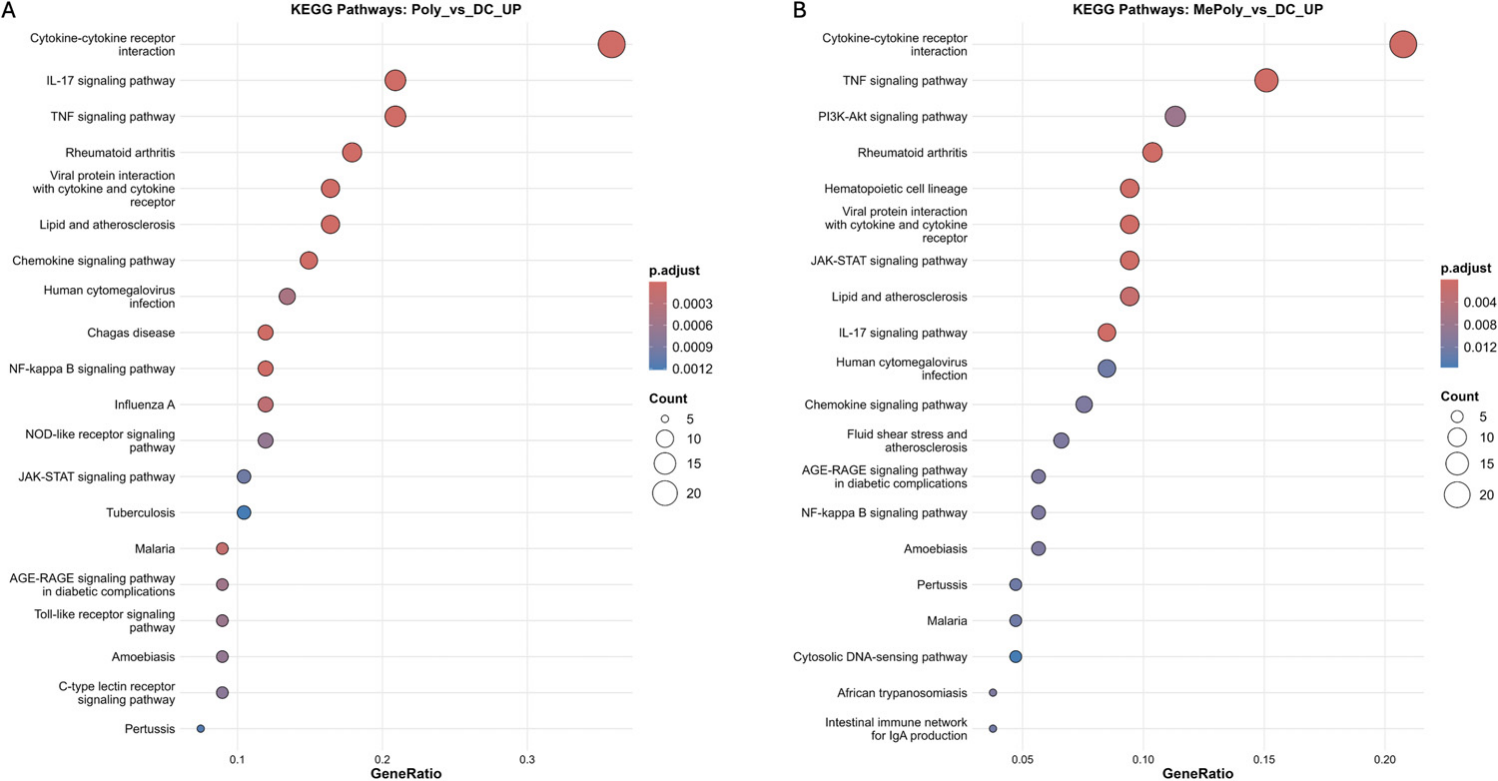
KEGG pathway enrichment analysis reveals key signaling cascades modulated by the various treatments. Dot plots displaying the top 20 enriched KEGG pathways for upregulated genes in the Poly vs Control (A) and 9MC6+Poly vs Control (B) groups. The x-axis shows the gene ratio (proportion of DEGs in each pathway), the dot size represents the gene count, and the dot color indicates statistical significance (p.adjust), with red denoting high significance.

The complete dataset includes quality control metrics (Table 1), DEG summaries (Table 2), hierarchical clustering analysis (Fig. 1), volcano plot visualization (Fig. 2), and functional enrichment results for Gene Ontology terms (Fig. 3) and KEGG pathways (Fig. 4).

**Table 1:** Summary of RNA-seq quality metrics and sample information.

Sample	Condition	Raw Reads (Millions)	Read Mapping (%)^a^	Gene Assignment (%)^b^	Gene Annotation Coverage (%)^c^	
					Org.Mn.eg.db	Biomart (Ensembl)
*DC*	*Control*	657.17	100	76.5	90.81	99.77
*Poly*	*Treated with* Poly I:C	648.72	100	69.5	90.78	99.77
*Me*	*Treated with* Poly I + C 9MC6	647.68	100	70.6	90.76	99.77

ᵃ Percentage of reads successfully aligned to mouse reference genome (GRCm39) by STAR aligner v2.7.10

ᵇ Percentage of mapped reads assigned to known gene exons by featureCounts v2.0.1

ᶜ Percentage of detected genes with successful symbol annotation from databases

**Table 2:** Summary of differentially expressed genes across three pairwise comparisons.

Comparison	Up-regulated DEGs	Down-regulated DEGs	Total DEGs
Poly(I:C) vs Control	184	13	197
9MC6+poly(I:C) vs Control	431	172	603
9MC6 vs Poly	139	166	305
**Total unique DEGs**			**826**

High-quality RNA sequencing was performed across all the samples, and comprehensive quality control metrics are presented in Table 1. Accordingly, raw sequencing generated approximately 650 million paired-end reads per sample, with 100% of the reads successfully mapped to the mouse reference genome (GRCm39), indicating excellent sequence quality and appropriate reference selection. Gene-level read assignment by featureCounts ranged from 69.5-76.5%, which is typical for mammalian RNA-seq where a substantial fraction of reads map to intergenic regions, multiple loci, or overlapping gene features. Gene annotation using Biomart/Ensembl achieved 99.77% coverage of detected genes, substantially higher than using the org.Mm.eg.db package (90.8% coverage), demonstrating the advantage of using up-to-date annotation resources.

Differential gene expression analysis across three pairwise comparisons identified 826 unique differentially expressed genes (DEGs) meeting the stringent criteria of FDR < 0.05 and |log2FC| > 1 (Fig. 1). The clustering dendrogram at the top of the heatmap shows that the two poly(I:C)-containing conditions (Poly and 9MC6+Poly) cluster together but remain distinct from each other, whereas the control sample forms a separate cluster, validating the biological relevance of the treatment effects.

Differential expression analysis revealed distinct transcriptional responses to poly(I:C) stimulation and modulation by 9MC6 treatment (Table 2). The poly(I:C) versus (vs) control comparison identified 197 DEGs (184 upregulated, 13 downregulated), predominantly representing proinflammatory genes and antiviral response factors. The 9MC6+poly(I:C) vs control comparison showed a more robust transcriptional response with 603 DEGs (431 upregulated, 172 downregulated), suggesting that 9MC6 enhances or modifies the cellular response to viral mimetic stimulation. Importantly, direct comparison between 9MC6+poly(I:C) and poly(I:C) conditions revealed 305 DEGs (139 upregulated, 166 downregulated), indicating specific gene expression changes attributable to 9MC6 treatment.

Volcano plot analysis provides a comprehensive view of the statistical significance and magnitude of expression changes for all detected genes across the three comparisons (Fig. 2). Top-ranked immune-related genes are labeled, including proinflammatory cytokines (*Il1a, Il1b, Ccl8, Cxcl3*, and *Serpinb2*), which are strongly upregulated, and downregulated genes (*Tlr4, Tespa1*, and *S100a8*). The distribution of points demonstrated robust transcriptional responses to poly(I:C) stimulation, with 9MC6 treatment inducing both enhancement and suppression of specific gene expression programs.

GO enrichment analysis of the upregulated genes revealed significant overrepresentation of immune-related biological processes across all the comparisons (Fig. 3).

Cytokine-related terms (cytokine activity, cytokine receptor binding, chemokine activity) dominated the upregulated genes, demonstrating strong activation of inflammatory signaling. 9MC6+Poly treatment exhibits broader functional enrichment including GTPase and growth factor receptor activities, whereas Poly treatment shows more focused enrichment of inflammatory response terms. The downregulated gene sets show enrichment of GTPase regulatory activities (B) and structural cellular components (D). Dot plots displaying the top 20 enriched KEGG pathways for each comparison. The cytokine-cytokine receptor interaction, TNF, IL-17 signaling pathway were the most prominently enriched pathways across all the conditions (Fig. 4).

## 4 Experimental Design, Materials and Methods

### 4.1 Preparation of 9-methoxycanthin-6-one

The compound 9-methoxycanthin-6-one used in this study was isolated from the hairy roots of *E. longifolia* as an amorphous yellow powder (200 mg). Its molecular formula, C₁₅H₁₀N₂O₂, was determined according to the extraction and structural elucidation procedures previously described in an our previous study [1]. Briefly, 9-methoxycanthin-6-one was isolated as an amorphous yellow powder. Its molecular formula was determined to be C₁₅H₁₀N₂O₂ on the basic of HR-ESI-MS analysis, which revealed a protonated molecular ion peak at *m/z* 251 [M+H]⁺. The ¹H-NMR spectrum exhibited characteristic signals of a canthin-6-one alkaloid. Aromatic proton signals were observed at *δ*_H_ 7.05 (1H, d, *J* = 8.5 Hz, H-10), 7.84 (1H, s, H-8), and 8.01 (1H, d, *J* = 8.5 Hz, H-11). In addition, two doublets at *δ*_H_ 8.08 (1H, d, *J* = 5.0 Hz, H-2) and 8.69 (1H, d, *J* = 5.0 Hz, H-1) were assigned to protons on a nitrogen-containing aromatic ring. Two further doublets at *δ*_H_ 6.88 (H-3) and 8.03 (H-4) corresponded to the protons of an aromatic ring bearing both carbon and nitrogen heteroatoms. The presence of a methoxy group was confirmed by a ¹³C-NMR signal at *δ*_C_ 55.7, along with a downfield aromatic carbon resonance at *δ*_C_ 161.6. The ¹³C-NMR and DEPT spectra revealed seven methine carbons and seven quaternary carbons, including a conjugated carbonyl carbon at *δ*_C_ 158.8. The spectrum of this compound is in agreement with the published spectrum of 9-methoxycathin-6-one [2,3].

### 4.2 Cell culture and treatments

The murine macrophage cell line, RAW 264.7 (ATCC TIB-71), was kindly provided by Dr. T. Kishimoto (Osaka University, Japan). The cells were cultured in RPMI 1640 medium (Sigma–Aldrich, St. Louis, MO, USA) supplemented with 10% fetal bovine serum (FBS), 100 µg/mL penicillin, and 100 µg/mL streptomycin (Corning Inc., Corning, NY, USA). The cells were seeded into 24-well plates at a density of 5 × 10⁴ cells per well. The compound 9-methoxycanthin-6-one was prepared as a stock solution in dimethyl sulfoxide (DMSO). Complete dissolution was achieved in 1.5 mL microcentrifuge tubes via gentle manual agitation in a 40°C water bath. For experimental treatments, RAW264.7 cells were preincubated with 30 µM of 9-methoxycanthin-6-one for 30 min, followed by stimulation with 20 µg/mL poly(I:C) (Merck, Darmstadt, Germany) [4]. The vehicle control group was treated with 0.1% (v/v) DMSO in the absence of poly(I:C) or 9-methoxycanthin-6-one. The culture flasks were incubated at 37°C in 5% CO₂. The cell pellets were collected 6 h after treatment.

### 4.3 RNA extraction and sequencing

Total RNA was extracted from treated and untreated RAW264.7 cells using a TRIzol reagent kit (Thermo Fisher Scientific, Waltham, MA, USA). RNA quantity and integrity were assessed using a Bioanalyzer (Agilent Technologies, Santa Clara, CA, USA), and pooled RNA from three experimental replicates was used for RNA-seq library preparation as previously described [5]. The preparation of sequencing libraries and paired-end sequencing were conducted by Novogene Co., Ltd. (Beijing, China). In summary, mRNA was captured using magnetic beads coated with poly-T oligonucleotides. The isolated mRNA was subsequently fragmented, and double-stranded cDNA was synthesized with random hexamer primers. The cDNA library was then constructed through a series of steps, including end repair, 3′ A-tailing, and adapter ligation. Afterward, the libraries underwent PCR amplification and purification. The purified libraries were pooled and subjected to sequencing on the Illumina NovaSeq 6000 platform (San Diego, CA, USA), yielding approximately 80–90 million paired-end reads per sample, each 150 bp in length.

### 4.4 Bioinformatics analysis

*Quality control:* Raw sequencing data were assessed using FastQC v0.12.1 with aggregate quality reports generated by MultiQC v1.29 [6]. The quality metrics included per-base quality scores, GC content, sequence duplication levels, and adapter contamination detection. Given the uniformly high quality (Q30+ scores throughout the read length), no quality trimming was performed to preserve maximum biological information, which is consistent with best practices for modern Illumina platforms.

*Read alignment:* Paired-end reads were aligned to the *Mus musculus* reference genome (GRCm39, Ensembl Release 112) using STAR aligner v2.7.11b [7] in two-pass mode. Key parameters: outSAMtype BAM SortedByCoordinate, quantMode GeneCounts for simultaneous counting.

*Gene quantification***:** featureCounts v2.0.1 [8] quantified reads at the gene level using Ensembl GTF annotation (Release 112) with the following parameters: paired-end mode, requiring both read ends mapped, excluding chimeric fragments, and counting only exonic reads.

*Differential expression***:** Differential gene expression (DEG) analysis was performed via the edgeR package (v3.38.4), a robust and widely cited method for RNA-seq count data analysis [9]. Low-expression genes were filtered (CPM > 1 in ≥1 sample). TMM normalization adjusted for library composition. Due to the single-sample design, the BCV was set to 0.4 (the standard for controlled in vitro experiments) [10]. Three pairwise comparisons were performed using exact test with Benjamini-Hochberg FDR correction. The significance criteria were as follows: FDR < 0.05, |log2FC| > 1.

*Functional annotation and data visualization***:** Biomart/Ensembl (Release 112) via biomaRt v2.52.0 achieved 99.77% gene annotation coverage. GO and KEGG enrichment analyses used the clusterProfiler v4.4.4 package with the org.Mm.eg.db v3.15.0 database, testing for overrepresentation among DEGs (FDR < 0.05). Hierarchical clustering heatmap was generated using pheatmap v1.0.12 with z-score normalization, Euclidean distance, and complete linkage. Volcano plots were created using ggplot2 v3.4.2 in R studio.

## Limitations

Because RNA from three biological replicates was pooled into single composite libraries per condition, this RNA-seq analysis provides descriptive insights rather than inferential statistics. Key gene expression changes were subsequently validated by qPCR to support the transcriptomic observations. Despite the utility of RAW 264.7 cells as a reproducible and robust model for evaluating the anti-inflammatory properties of 9-methoxycanthin-6-one, certain biological constraints must be acknowledged. Compared with primary macrophages, RAW 264.7 macrophages may exhibit altered signaling kinetics. Therefore, these results should be further validated in primary cell models or *in vivo* systems to confirm their broader physiological relevance.

## Ethics Statement

The authors have read and follow the ethical requirements for publication in Data in Brief and confirm that the current work does not involve human subjects, animal experiments, or any data collected from social media platforms.

## CRediT Author Statement

**Trang Thu Tran**: Sampling, Methodology, Investigation, Validation, Writing - Original Draft, Writing - Review & Editing; **Huyen Minh Thi Ta**: Sampling, Methodology, Formal analysis, Data curation, Validation, Writing - Original Draft, Writing - Review & Editing; **Duc Hoang Le**: Software, Formal analysis, Data Curation, Writing - Original Draft, Writing - Review & Editing; **Duong Huy Nguyen**: Software, Formal analysis, Data Curation, Writing - Review & Editing; **Nam Trung Nguyen**: Supervision, Project administration, Funding acquisition, Writing- Original Draft; Writing - Review & Editing.

## Data Availability

NCBITranscriptome dataset of murine macrophages from Mus musculus (Original data)
